# Genetic Characterisation of Feeding Patterns in Lactating Holstein Cows and Their Association With Feed Efficiency Traits

**DOI:** 10.1111/jbg.12911

**Published:** 2024-11-11

**Authors:** Ligia Cavani, Kristen L. Parker Gaddis, Ransom L. Baldwin, José E. P. Santos, James E. Koltes, Robert J. Tempelman, Michael J. VandeHaar, Heather M. White, Francisco Peñagaricano, Kent A. Weigel

**Affiliations:** ^1^ Department of Animal and Dairy Sciences University of Wisconsin Madison Wisconsin USA; ^2^ Council on Dairy Cattle Breeding Bowie Maryland USA; ^3^ Animal Genomics and Improvement Laboratory, ARS, USDA Beltsville Maryland USA; ^4^ Department of Animal Sciences University of Florida Gainesville Florida USA; ^5^ Department of Animal Science Iowa State University Ames Iowa USA; ^6^ Department of Animal Science Michigan State University East Lansing Michigan USA

**Keywords:** area under the curve, heritability, intake, milk energy

## Abstract

Feeding behaviour traits, such as number, duration or intake per feeder visit, have been associated with feed efficiency in dairy cattle. Those traits, however, do not fully capture cows' feeding patterns throughout the day. The goal of this study was to propose a new phenotype for characterising within‐day feeding patterns and estimate its heritability and genetic correlations with dry matter intake (DMI), secreted milk energy, metabolic body weight and residual feed intake. Feeding patterns were evaluated using 4.8 million bunk visits from 1684 midlactation Holstein cows collected from 2009 to 2023 with an Insentec system. Feed efficiency traits were available from 6099 lactating Holstein cows at six research stations across the United States. Daily bunk visits were ordered, with Time 0 designated as the time of first feed delivery. Intake proportions were calculated by visit for each cow by dividing feed intake per visit by the total intake of the cow for that day. Feeding patterns were characterised by the area under the curve of cumulative feed intake proportions for each cow throughout the day. The feeding pattern phenotype per cow was defined as the average of areas under the curve across days, whereas consistency of feeding pattern was calculated as the natural logarithm of variance of daily area under the curve values. Estimates of heritability and genetic correlations were performed using Bayesian inference with an animal model, considering lactation, days in milk and cohort (trial–treatment) as fixed effects and animal as a random effect. Heritability estimates for average area under the curve and variance of daily area under the curve were 0.35 ± 0.05 and 0.16 ± 0.05, respectively. The genetic correlation between average area under the curve and secreted milk energy was −0.30 ± 0.14. Genetic correlations between average area under the curve and DMI, metabolic body weight and residual feed intake were not statistically significant. Variance of daily area under the curve was genetically correlated with DMI (0.47 ± 0.15), secreted milk energy (0.40 ± 0.17) and metabolic body weight (0.28 ± 0.13). The genetic correlation between variance of daily area under the curve and residual feed intake was not significant. Overall, we provided a reliable method to truly characterise feeding patterns in midlactation dairy cows. Feeding pattern and its consistency were heritable, indicating that a significant proportion of phenotypic variation is explained by additive genetic effects. Genetic correlation estimates indicate that cows with more consistent daily feeding patterns have lower DMI, lower secreted milk energy and lower metabolic body weight.

## 1 Introduction

Feeding behaviour has been associated with important traits related to feed efficiency, welfare and production in dairy cattle (DeVries 2019; Brown et al. 2022; Cavani et al. 2022; Reyes et al. 2023), as well as beef cattle (Benfica et al. 2020; Koenig et al. 2020; Kava et al. 2023), broilers (Howie et al. 2011; Yan et al. 2019) and pigs (Kavlak and Uimari 2019; Fornós et al. 2022; He et al. 2022). Interestingly, there is growing evidence that feeding behaviour is an important contributor to genetic differences in feed efficiency among lactating cows, and behavioural data could be used to enhance the reliabilities of genetic evaluations for feed efficiency and accelerate genetic progress (Cavani et al. 2022).

In general, feeding behaviour has been analysed using traits such as number of feeder visits per day, duration per visit, intake per visit and feeding rate. However, these traits do not fully capture the trajectory of cows' feeding patterns throughout the day, especially their behaviour relative to the time of feed delivery. In modern intensive dairy farm systems, the delivery of fresh feed has a substantial impact on feeding behaviour, primarily by stimulating feeding activity (DeVries et al. 2003; DeVries and Von Keyserlingk 2005). Indeed, Brown et al. (2022) observed an increase in dry matter intake (DMI), bin visits and feeding rate after the first feed delivery in midlactation Holstein cows.

**FIGURE 1:**
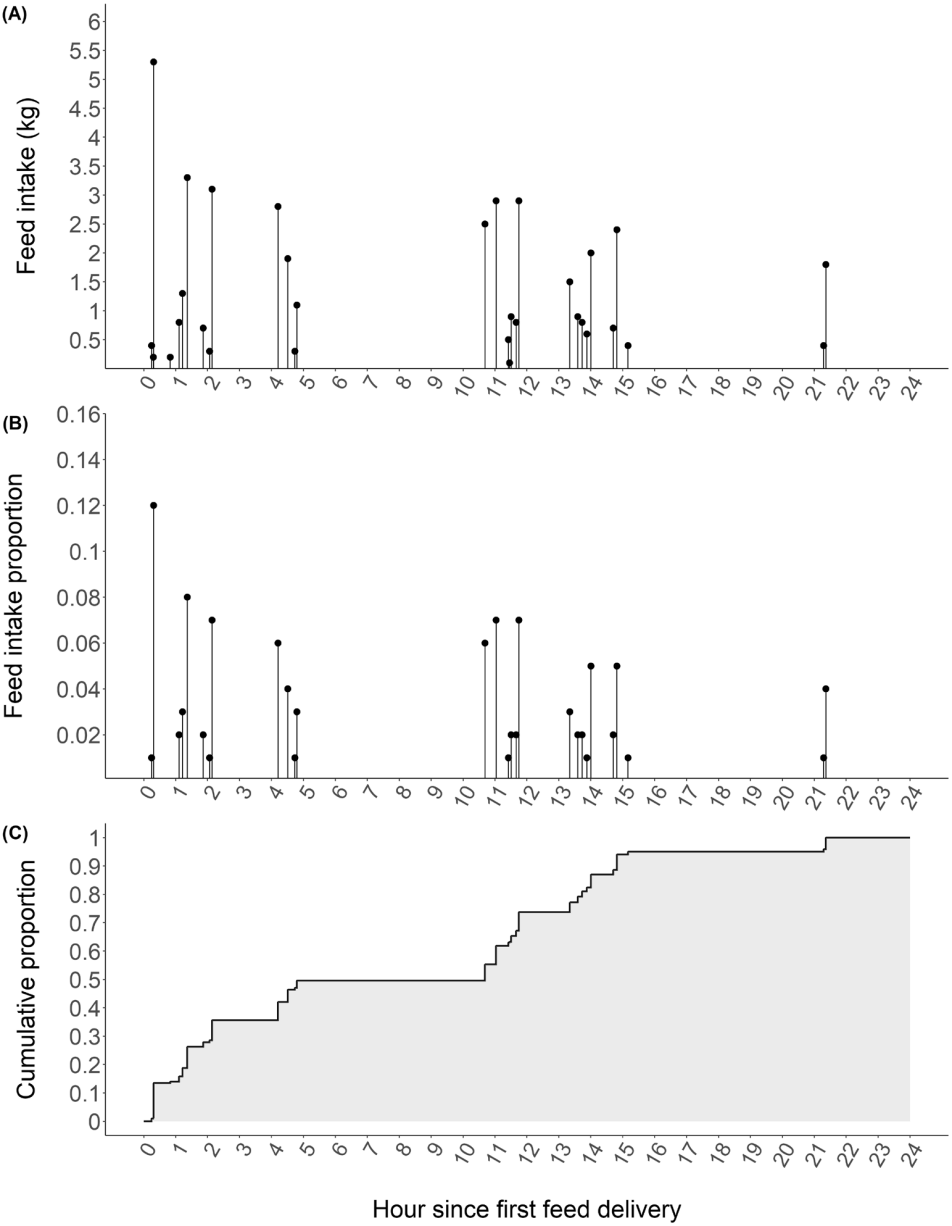
Example of the method used to characterise feeding patterns for a cow with an average number of bin visits and feed intake in 1 day. (A) Feed intake (kg) per bin visit, (B) feed intake proportions calculated by dividing feed intake (kg) of each visit by the total intake (kg) of that day and (C) cumulative feed intake proportions, with the area under the curve depicted in grey.

The objective of this study was to propose a new method for characterising feeding patterns in dairy cows by measuring how cows distribute their total intake throughout the day relative to time of first feed delivery (Figure 1). Additionally, our goals were to estimate genetic parameters of daily feeding pattern and its consistency across days, and estimate genetic correlations with feed efficiency traits, namely, DMI, secreted milk energy (MilkE), metabolic body weight and residual feed intake (RFI).

## 2 Materials and Methods

### 2.1 Feeding Patterns: Data

Feeding patterns were evaluated using feeding records from 1684 lactating Holstein cows (615 primiparous and 1069 multiparous) collected from 2009 to 2023 in a free‐stall facility at the University of Wisconsin‐Madison Emmons Blaine Dairy Cattle Research Center (Arlington, WI) with a roughage intake control system (RIC; Hokofarm Group). The RIC system permits one animal to access the feeder at a given time and records the start and end time of each visit to the feeder and the weight of feed consumed during each visit. Cows (*n* = 64 maximum in the pen) typically had access to all 32 feeders. The raw data generated by this system consisted of 4.8 million feeder visits and included date, time entered the feeder, time exited the feeder, weight of feed at entry, weight of feed at exit and identification number of transponder, cow and feeder. For those cows that participated in multiple trials, only data from the first trial were used. Records from feeder visits with as‐fed intake ≤ 0 or > 20 kg or visit duration < 5 s or > 3000 s were removed.

### 2.2 Feeding Patterns: Characterisation and Measurements

Cows were fed twice a day, typically at 11:00 ± 1 h and 17:00 ± 1 h. We were able to determine the time of first feeding precisely because the cows were locked away from the bins to discard orts and distribute fresh feed. After determining the time of the first feed delivery, the subsequent steps for characterising feeding patterns were as follows: (1) order the bunk visits by ascending time (hour:minute:second) across all cows in the pen; (2) designate the time of the first bunk visit by any cow after the lockout period for fresh feed delivery as Time 0; (3) calculate the proportion of feed consumed at each bunk visit by dividing feed intake (kg) of the visit by total intake (kg) of the cow for that day; (4) translate feed intake proportions into cumulative proportions, from the time of fresh feed delivery on a given day until the lockout period on the subsequent day; and (5) compute the area under the curve of cumulative feed intake proportions for each cow on each day using the trapezoid method.

Data cleaning involved excluding days in which a cow visited the feeder fewer than 5 times, and days with total intake of ≤ 12 or > 115 kg. Furthermore, within each cow, records were removed if the area under the curve exceeded 3.5 standard deviations from the cow's mean area under the curve over the entire period. The final dataset of feeding patterns comprised 1684 cows, with an average of 53 days per cow, and a range from 22 to 107 days.

The feeding pattern phenotype for each cow was defined as the average of the areas under the curve for her experimental period (AUC). We also evaluated consistency of feeding pattern, which was calculated as the natural logarithm of variance of daily area under the curve (log‐Var‐dAUC). The log transformation of the variances was implemented due to normality assumptions of the models used in this study.

The methodology for characterising feeding patterns is depicted in Figure 1, showing real data for a cow on a specific day. This cow visited the bin 31 times with a total feed intake of 43.8 kg corresponding to average values in our dataset. After the first feed delivery, which occurred at 11:20 PM on that day and is represented as Hour 0 in the figure, the feed intake for the first, second and third bin visits were 0.4, 0.2 and 5.3 kg, respectively (Figure 1A). These values represented proportions of 0.009, 0.005 and 0.13, respectively, of the cow's total feed intake for that day (Figure 1B). By translating the feed intake proportions into cumulative proportions, we can calculate the area under the curve (Figure 1C), which was used to quantify the feeding pattern.

**FIGURE 2:**
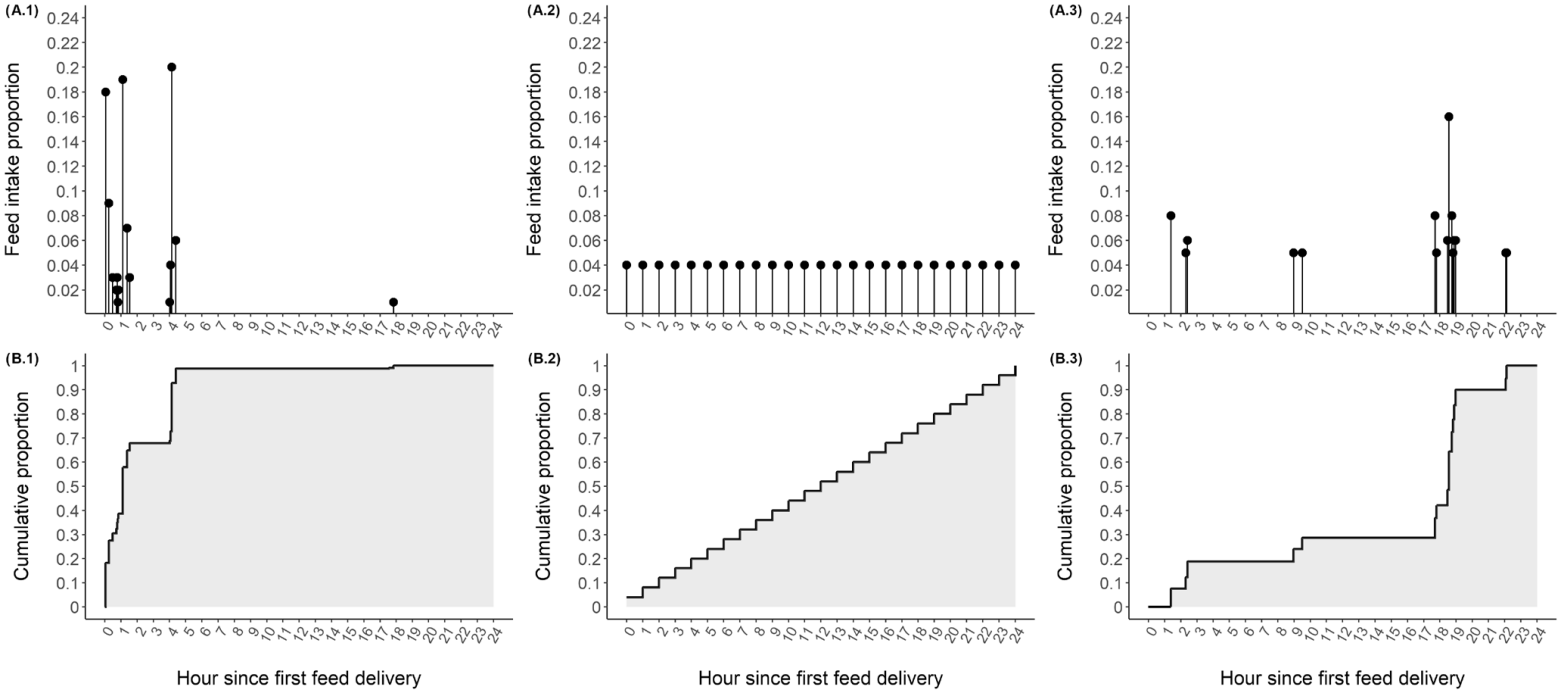
Feeding pattern characterisation: Examples of three cows: (1) real data of a cow with high area under the curve of cumulative feed intake proportions in 1 day, (2) simulated data for a cow with a uniform feeding pattern throughout the day and (3) real data of a cow with low area under the curve of cumulative feed intake proportions in 1 day. (A) and (B) represent the feed intake proportions of each bin visit, and the cumulative feed intake proportions with the area under the curve depicted in grey, respectively.

Additional examples of using area under the curve of the cumulative feed intake proportions to quantify feeding patterns are illustrated in Figure 2. In an overly simplistic case, the cow's total intake for the day would be equally and uniformly divided among bin visits over 24 h, with the same amount of feed consumed in each visit (Figure 2A.2), resulting in an area under the curve of the cumulative feed intake proportions equal to 43,200 (half a day, in seconds; Figure 2B.2). However, in reality, a cow may consume most of its total intake in the first few hours after feed delivery (Figure 2A.1,B.1) or she may delay much of her feeding until later in the day (Figure 2A.3,B.3), leading to different feeding patterns and area under the curve metrics.

**FIGURE 3:**
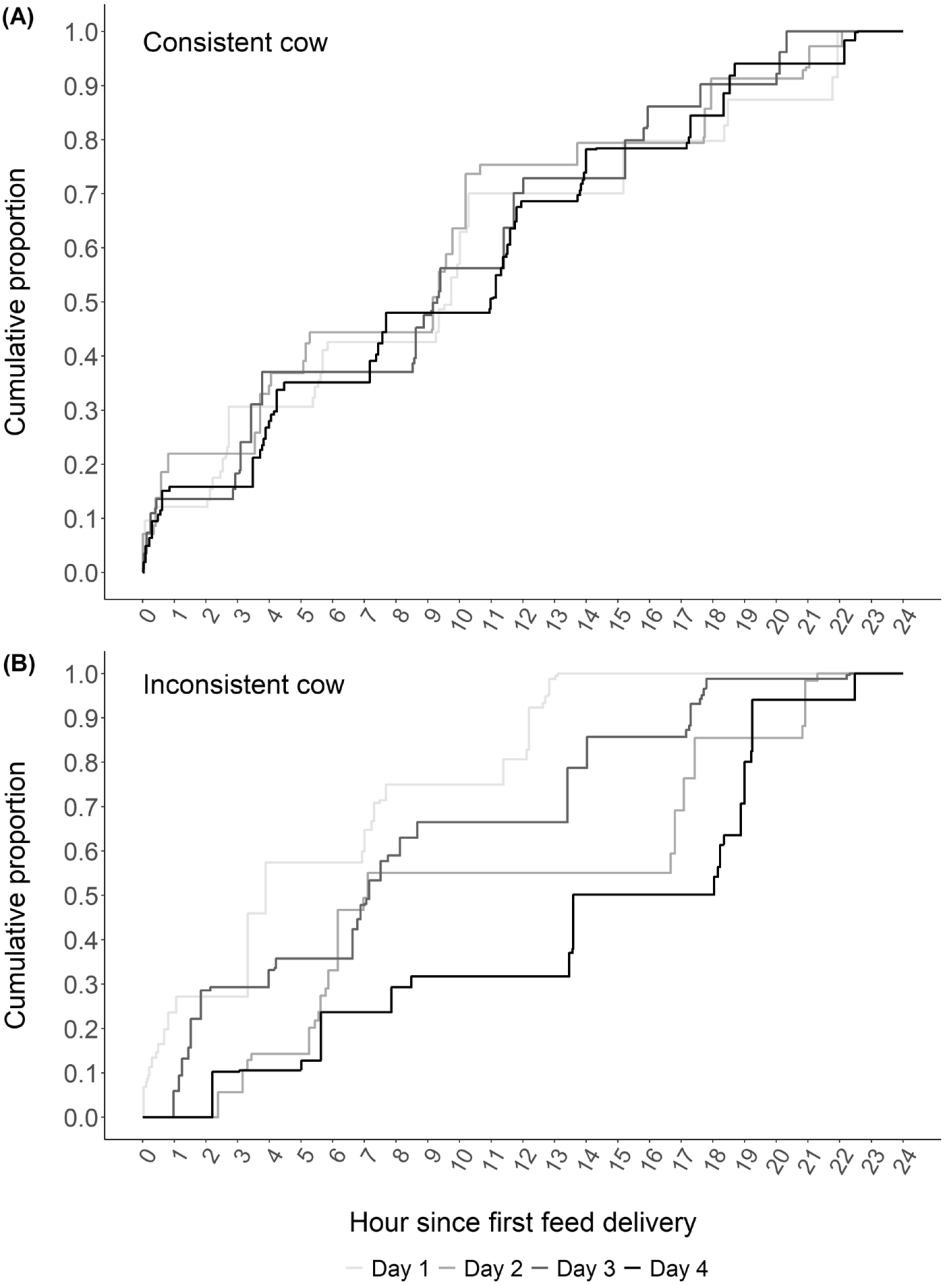
Example of feeding pattern consistency of two cows on 4 different days. (A) Consistent cow and (B) inconsistent cow.

The consistency of feeding patterns across days is illustrated in Figure 3 using data from two example cows in this study. A consistent cow exhibited similar feeding patterns on different days, while an inconsistent cow showed variation in feeding patterns, resulting in low and high day‐to‐day variations in area under the curve, respectively.

### 2.3 Feed Efficiency: Data and Traits

Feed efficiency traits were evaluated using data from 6099 lactating Holstein cows collected from 2007 to 2023 at six research stations: Iowa State University (Ames, IA; 1332 cows), Michigan State University (East Lansing, MI; 525 cows), University of Florida (Gainesville, FL; 936 cows), University of Wisconsin‐Madison (Arlington and Marshfield, WI; 1934 cows), USDA‐Agricultural Research Service Animal Genomics and Improvement Laboratory (Beltsville, MD; 823 cows) and USDA‐Agricultural Research Service U.S. Dairy Forage Research Center (Madison, WI; 549 cows). The 1684 cows with feeding behaviour data represented a subset of the 6099 cows with feed efficiency data. All procedures were approved by the corresponding Institutional Animal Care and Use Committees. Animals were housed in free‐stall or tie‐stall facilities, and daily feed intakes were measured via Roughage Intake Control System (Hokofarm Group, Emmeloord, Flevoland, The Netherlands), Calan Broadbent Feeding System (American Calan, Northwood, New Hampshire, USA), GrowSafe System (Vytelle, Lenexa, Kansas, USA) or manual weigh‐back of refusals. In addition, milk yield, milk composition and BW were recorded. Details of data collection protocols varied by station, but generally DMI and milk yield data were measured daily, milk components were analysed at least once per week and BW were measured daily, weekly or on multiple consecutive days at the beginning, middle and end of each trial (Tempelman et al. 2015; Cavani et al. 2023). For those cows that participated in multiple trials, only data from the first trial were used.

The following feed efficiency traits were considered for each cow: DMI, MilkE, metabolic BW and RFI. Descriptive statistics for all traits are shown in Table 1. MilkE was calculated by week using the following equation and then averaged by cow to obtain the MilkE per individual.

$$\begin{array}{cc} \text{MilkE}\;\left(\text{Mcal}\right)=\; & \left(0.0929\times \mathrm{fat}\%+0.0563\times \text{protein}\%+0.0395\times \text{lactose}\%\right)\\ & \times \text{milk yield}\;\left(\mathrm{kg}\right). \end{array}$$

**TABLE 1:** Descriptive statistics for feeding pattern, consistency of feeding pattern and feed efficiency traits in lactating Holstein cows.

Traits	No. of cows	Mean	SD	Minimum	Maximum
Feeding pattern					
AUC	1684	56,345	2279.6	48,691	66,059
Consistency of feeding pattern					
log‐Var‐dAUC	1684	16.94	0.35	15.80	18.06
Feed efficiency traits					
DMI (kg)	6099	24.80	3.92	10.80	44.53
Secreted milk energy (Mcal)	6099	29.56	5.46	8.91	49.25
Metabolic body weight (kg^0.75^)	6099	125.7	11.9	94.9	176.2
Residual feed intake (kg)	6099	0	1.63	−10.39	10.22

Abbreviations: AUC, average area under the curve of daily cumulative feed intake proportions; log‐Var‐dAUC, natural logarithm of variance of daily area under the curve.

Similarly, BW were recorded manually on at least 3 consecutive days at the beginning, middle and end of the trial period in most studies, whereas other studies recorded BW manually on a weekly basis, and others recorded BW daily using walk‐over scales. We used linear regression of measured BW on day of trial to estimate missing BW records. Metabolic BW was calculated as the cow's average BW^0.75^.

RFI for each cow was calculated using the following linear mixed model, DMI = DIM + Lact + Cohort + *b*_*1*_MilkE + *b*_*2*_mBW + *b*_*3*_ΔBW + e, where DIM represents the fixed effect of days in milk with nine levels (grouped by 16‐day periods: 50–66, 67–83, 84–100, 101–117, 118–134, 135–151, 152–168, 169–185 and > 186), Lact represents the fixed effect of lactation number with four levels (1, 2, 3 and 4+), Cohort represents the fixed effect of trial–treatment with 173 levels, MilkE is secreted milk energy with partial regression coefficient *b*_1_, mBW is metabolic BW with partial regression coefficient *b*_2_, ΔBW is change in BW (difference in BW at the end and beginning of each trial period) with partial regression coefficient *b*_*3*_ and e is the random residual of the model assumed to follow a normal distribution *e* ~ *N*($0,I\sigma_{e}^{2}$). This residual represents the RFI phenotype used in the genetic analysis described below.

### 2.4 Genetic Analyses

Estimates of heritability for AUC and log‐Var‐dAUC traits were performed using Bayesian inference with an individual animal model. Relationships were traced back for five generations. Systematic (fixed) effects included lactation number with four levels (1 to 4+), midpoint days in milk with nine levels (grouped by 16 days) and cohort effect of trial–treatment.

Estimates of genetic correlations between AUC and feed efficiency traits, as well as between log‐Var‐dAUC and feed efficiency traits, were also performed using Bayesian inference. Bivariate models included the same systematic (fixed) effects used in the univariate models except for RFI. The systematic (fixed) effects of lactation number, days in milk and cohort were not included in the genetic model for RFI, as they had already been considered in the calculation of the RFI phenotype. The random effects followed a joint distribution as:

$$\left[\begin{array}{c} u\\ e \end{array}\right]\sim N\left\{\left[\begin{array}{c} 0\\ 0 \end{array}\right],\left[\begin{array}{cc} G_{0}⊗A & 0\\ 0 & R_{0}⊗I \end{array}\right]\right\},$$

where u and e represent the additive genetic and residual effects, respectively; **G**_**0**_ is the additive genetic direct effects (co)variance matrix; **A** is the matrix of additive relationships between animals in the pedigree of the last five generations; **R**_**0**_ is the 2 × 2 residual (co) variance matrix; and **I** is an identity matrix with suitable dimensions.

Variance and covariance components were estimated using the gibbsf90+ software from the BLUPF90 family of programs (Aguilar et al. 2018). The default prior distributions were used, that is, flat distribution for systematic effects, normal distribution for random effects, scaled inverse Chi‐square for single trait variance component and Wishart for bivariate variance components. A Gibbs sampling chain with 1,000,000 samples was generated, with burn‐in and thin parameters equal to 200,000 and 10, respectively. The thin parameter was considered only for the bivariate analyses. Convergence was analysed based on visual inspection of trace plots and the convergence tests of Gelman an Rubin (1992) and Geweke (1986) as well as of Heidelberger and Welch (1981), using the coda package of the R software (Plummer et al. 2006).

## 3 Results and Discussion

Posterior means of additive genetic variances, residual variances and heritability estimates for AUC and log‐Var‐dAUC are shown in Table 2. Both feeding pattern and consistency of feeding pattern are heritable traits. The heritability estimate for AUC was 0.35 ± 0.05, whereas the heritability estimate for log‐Var‐dAUC was 0.16 ± 0.05.

**TABLE 2:** Posterior mean (±SD) and 90% highest posterior density intervals (within parentheses) of variance component and heritability estimates for feeding patterns (AUC) and consistency of feeding pattern (log‐Var‐dAUC) in lactating Holstein cows.

Parameter	AUC	log‐Var‐dAUC
Additive genetic variance	1,315,780 ± 250,145	0.017 ± 0.006
	(940,700; 1,685,000)	(0.008; 0.025)
Residual variance	2,421,331 ± 189,574	0.087 ± 0.005
	(2,108,000; 2,719,000)	(0.078; 0.095)
Heritability	0.35 ± 0.05	0.16 ± 0.05
	(0.26; 0.44)	(0.08; 0.24)

Abbreviations: AUC, average area under the curve of daily cumulative feed intake proportions; log‐Var‐dAUC, natural logarithm of variance of daily area under the curve.

Posterior means of genetic correlation between feeding pattern and feed efficiency traits, as well as genetic correlations between consistency of feeding pattern and feed efficiency traits, are shown in Table 3. Feeding pattern, measured as AUC, was negatively correlated with DMI, MilkE and RFI, and positively correlated with mBW. Genetic correlations between consistency of feeding pattern, measured as log‐Var‐dAUC, and feed efficiency traits ranged from 0.24 to 0.47.

**TABLE 3:** Posterior mean (±SD) and 90% highest posterior density intervals (within parentheses) of genetic correlation estimates between feeding patterns (AUC) and feed efficiency traits, and between consistency of feeding pattern (log‐Var‐dAUC) and feed efficiency traits in lactating Holstein cows.

	AUC	log‐Var‐dAUC
DMI^a^	−0.18 ± 0.12	0.47 ± 0.15
	(−0.39; 0.02)	(0.21; 0.66)
Secreted milk energy^a^	−0.30 ± 0.14	0.40 ± 0.17
	(−0.52; −0.07)	(0.05; 0.64)
Metabolic body weight^a^	0.16 ± 0.10	0.28 ± 0.13
	(0.00; 0.33)	(0.08; 0.49)
Residual feed intake^a^	−0.18 ± 0.14	0.24 ± 0.14
	(−0.41; 0.05)	(0.01; 0.44)

Abbreviations: AUC, average area under the curve of daily cumulative feed intake proportions; log‐Var‐dAUC, natural logarithm of variance of daily area under the curve.

^a^ Heritability estimates (±SD): DMI = 0.38 ± 0.04; secreted milk energy = 0.32 ± 0.03; metabolic body weight = 0.59 ± 0.04; residual feed intake = 0.29 ± 0.03.

Ruminants exhibit diurnal and pulsative patterns as a part of natural feeding behaviour. Despite variation caused by factors such as pasture quality and temperature, grazing lactating cows present two main peaks of eating activity: morning and evening (Van Soest, 1994). In modern intensive dairy farm systems, including free‐stall facilities, feeding activity peaks after fresh feed delivery (DeVries et al. 2003; DeVries and Von Keyserlingk 2005; Brown et al. 2022). These systems, which typically have two feed deliveries per day, exhibit pulsative patterns like those observed in natural grazing systems, with a larger peak of eating activity right after the first feed delivery in the mornings. However, it has been demonstrated that daily feeding patterns of group‐housed dairy cows kept indoors are influenced more heavily by the timing of fresh feed delivery than by the time of day (DeVries and Von Keyserlingk 2005). In this study, we used data from automated feeders in a free‐stall facility to investigate feeding patterns over a 24‐h period using time of first feed delivery as a starting point to characterise the feeding pattern. To our knowledge, this is the first study to propose the area under the curve of daily cumulative feed intake proportions as a phenotype for assessing daily feeding patterns in livestock species.

Traditional feeding behaviour traits, such as number, duration or intake per feeder visit, explain part of the observed variation in feed efficiency among lactating cows; however, these traits do not reflect daily feeding patterns. For example, cows with the same number of bin visits and daily feed intake could have totally different feeding patterns, as shown in Figure 2. Applying the method proposed in this study, these two example cows will have widely different AUC values due to different proportional consumption of DMI throughout the day. Previous studies in which feeding behaviour was analysed using daily blocks of 2 or 3 h in Holstein cows and heifers (Green et al. 2013; Brown et al. 2022) provided important insights into differences in feeding behaviour at certain times of the day, but they reflected feeding patterns during a small proportion of the day. In pigs, a new method of describing individual diurnal patterns has been proposed based on the detection of circadian rhythms (Bus et al. 2023), and the authors noted that individual animals differed in their diurnal patterns of feed intake.

Our study showed that feeding pattern is heritable, indicating that a substantial proportion of phenotypic variance is explained by additive genetic effects, such that AUC could be incorporated into genetic selection schemes. Additionally, consistency of feeding pattern is moderately heritable, suggesting that genetic selection for log‐Var‐dAUC may effectively reduce variation in cow feeding patterns across different days, which could enhance the operational efficiency of feeding routines on the farm. Daily feeding pattern in the present study showed similar heritability as feeding behaviour in our previous study, where we reported heritability estimates from 0.19 to 0.32 for weekly averages of traits such as number of feeder visits per day, duration of each feeder visit, intake per each feeder visit and feeding rate Cavani et al. (2022).

Feeding patterns were, in general, not significantly genetically correlated with feed efficiency traits. Nonetheless, larger AUC was genetically associated with lower MilkE (genetic correlation of −0.30 ± 0.14; highest posterior density intervals −0.52 to −0.07), suggesting that those cows which consume most of their total daily intake in the first few hours after feed delivery (larger AUC) tend to produce less milk. Consistency of feeding pattern, measured as log‐Var‐dAUC, was significantly genetically correlated with DMI, MilkE and mBW. Higher variance of daily area under the curve was genetically associated with higher DMI (genetic correlation of 0.47 ± 0.15; highest posterior density intervals 0.21–0.66), higher MilkE (genetic correlation of 0.40 ± 0.17; highest posterior density intervals 0.05–0.64) and greater metabolic body weight (genetic correlation of 0.28 ± 0.13; highest posterior density intervals 0.08–0.49).

This further indicates that genetic selection of cows with more consistent daily feeding patterns could potentially favour the selection of more feed‐efficient cows in future generations. Interestingly, studies in beef cattle and pigs found similar associations between greater feed efficiency and less day‐to‐day variation in traits such as number of feeder visits and daily bunk time (Putz et al. 2019; Parsons et al. 2020; Homma et al. 2021). Moreover, it was revealed that lactating Holstein cows with greater variation in daily DMI (i.e., less consistency) are less feed efficient and may be less resilient (Cavani et al. 2023).

Data from automated feeding systems installed in research stations, utilised for individual feed intake measurements, offer a valuable opportunity to analyse feeding patterns in lactating Holstein cows. Our study introduced a novel method to characterise feeding patterns in dairy cows, providing new insights into their genetic variability and their genetic associations with feed efficiency traits. As a novel trait, we used Bayesian approach to conduct the genetic analyses due to advantages this method provides regarding estimate uncertainties, as all results are derived from probability distributions (Gianola and Rosa 2015). As a first study, we provided a reliable method to truly characterise feeding patterns in lactating cows, and it brings new insights into how feeding patterns may explain differences in feed efficiency among cows. However, despite the unique dataset provided by the automated feeding system used in this study, future studies applying our method with a larger number of cows would be important to validate our findings. Additionally, our method could be implemented using computer vision systems based on cameras, enabling the collection of feeding pattern phenotypes in a greater number of cows.

## 4 Conclusions

In this study, a new method was proposed to characterise daily feeding patterns in midlactation dairy cows, and we investigated the genetic variation in daily feed intake trajectory and the association with feed efficiency. Feeding pattern was heritable, indicating that a significant proportion of phenotypic variation is explained by additive genetic effects. Similarly, consistency of feeding patterns was also heritable, suggesting that genetic selection could reduce variation in cow feeding patterns across different days, which may contribute to an enhanced feeding routine on the farm. Genetic correlation estimates indicate that cows with more consistent daily feeding patterns have lower DMI, lower MilkE and lower metabolic body weight.

## Conflicts of Interest

The authors declare no conflicts of interest.

## Data Availability

The data that support the findings of this study are available from the corresponding author upon reasonable request.
